# Brain activation patterns of figurative language comprehension in individuals with autism spectrum disorder: an activation likelihood estimation meta-analysis

**DOI:** 10.3389/fnins.2026.1717020

**Published:** 2026-01-30

**Authors:** Lulu Cheng, Jianxin Zhang, Haoran Mao, Xize Jia, Linlin Zhan, Cuicui Liu

**Affiliations:** 1 School of Foreign Studies, China University of Petroleum (East China), Qingdao, China; 2 School of Psychology, Zhejiang Normal University, Jinhua, China; 3 Key Laboratory of Intelligent Education Technology and Application of Zhejiang Province, Zhejiang Normal University, Jinhua, China; 4 School of Foreign Languages and Literature (School of Region and Country Studies), Heilongjiang University, Harbin, China; 5 Pediatric Department, Qingdao Huangdao Distinct People’s Hospital, Qingdao, China

**Keywords:** activation likelihood estimation, autism spectrum disorder, figurative language comprehension, functional magnetic resonance imaging, neuroimaging

## Abstract

**Background:**

Individuals with Autism Spectrum Disorder (ASD) consistently exhibit difficulties in comprehending figurative language. While prior neuroimaging studies have identified discrepancies in brain activation between ASD individuals and neurotypical controls (NCs) during such tasks, the specific and consistent neural patterns underlying these deficits remain unclear.

**Methods:**

We conducted an activation likelihood estimation (ALE) meta-analysis to identify consistent brain activation patterns associated with figurative language comprehension in ASD. A systematic literature search was performed across five databases (PubMed, Embase, Web of Science, CNKI, Wanfang) up to January 31, 2025. Six functional magnetic resonance imaging (fMRI) studies, comprising 95 individuals with ASD and 98 NCs, met the inclusion criteria.

**Results:**

The analysis revealed that during figurative language comprehension, both ASD and NCs consistently activated a core network involving the bilateral superior temporal gyrus (STG), transverse temporal gyrus, and right insula. A conjunction analysis confirmed the stability of these shared regions. Crucially, the meta-analysis of group differences (NCs > ASD) identified a significant cluster of hypoactivation in the ASD group within the left STG and middle temporal gyrus (MTG). No significant hyperactivation was found in the ASD group compared to NCs.

**Conclusion:**

This meta-analysis demonstrates that while individuals with ASD recruit typical language networks during figurative language comprehension, they exhibit a consistent pattern of reduced neural recruitment in the left STG and MTG. This hypoactivation may reflect a dual deficit encompassing reduced efficiency in semantic access/integration and impaired socio-linguistic integration, providing a neurobiological substrate for the pragmatic language difficulties characteristic of ASD.

**Systematic review registration:**

https://www.crd.york.ac.uk/PROSPERO/view/CRD42023467185, CRD42023467185.

## 1 Introduction

Autism Spectrum Disorder (ASD) is a complex neurodevelopmental condition, characterized by core features including deficits in social communication and interaction, alongside restricted and repetitive behaviors and interests (American Psychiatric Association, 2013). Although the debate surrounding the relationship between ASD and core linguistic skills has persisted for several years, a consensus among numerous studies indicates that individuals with ASD universally encounter difficulties in understanding and using language in social context (Kalandadze et al., 2018). Among the primary pragmatic deficits, the challenge in processing figurative language is particularly pronounced, which is considered to be one of the characteristics of ASD (Lampri et al., 2024). Figurative language refers to a class of linguistic expressions, such as metaphor, irony, idioms and sarcasm, whose interpretation is inherently nonliteral, where the conveyed meanings of words, phrases or sentences diverge from their literal ones (Kalandadze et al., 2018; Lampri et al., 2024; Vulchanova et al., 2015). Thus, to comprehend figurative language, individuals need to discern the speaker’s intentions within a specific context. Although figurative language is a deviation from literal meaning, it is a common phenomenon in daily language and social communication (Kalandadze et al., 2018; Xu and Cao, 2021). For instance, in conversations among friends, approximately 8% of adult speech contains some degree of irony (Gibbs, 2000). Teachers frequently employ idioms when instructing students, with an average of 1.73 idioms per minute, and popular children’s books contain a significant amount of complex metaphorical language, with an average of 54 metaphors per 1,000 words (Colston and Kuiper, 2002). In other words, figurative language is not confined to a singular rhetorical device only to be utilized in unusual conditions. Individuals adeptly use a variety of figurative language to articulate a broad spectrum of both blatant and subtle interpersonal meanings. Consequently, the ability to comprehend figurative language is not only an indication of language proficiency, but also a crucial factor for successful social interaction, inferring others’ intentions, and emotional states (Thoma et al., 2006). Difficulties in this domain can directly exacerbate social isolation and integration challenges for individuals with ASD, who exhibit core deficits in social communication (Kalandadze et al., 2018). Therefore, figurative language comprehension impairment in autistic individuals has attracted the attention of scholars.

Previous research on the comprehension of figurative language in individuals with ASD has predominantly conducted from behavioral or cognitive perspectives. For example, Happé (1993) confirmed that the theory of mind (ToM) was vital for the comprehension of metaphor, simile and irony. By exploring the impact of ToM and language ability on metaphor comprehension, Norbury (2005) suggested that language skills, particularly semantic ability, are more critical for metaphor comprehension than ToM skills alone. It can be seen that the explanations for deficits in understanding figurative language among autistic individuals have been extensively studied. While these behavioral and cognitive studies offer crucial insights into the figurative language deficits in ASD, the stable neural substrates underlying these difficulties remain unclear. Identifying consistent neurobiological patterns is essential for bridging heterogeneous cognitive phenotypes with brain-level dysfunctions, and for clarifying whether figurative language impairments in ASD are associated with reliable alterations in core language-social networks (Chen and Glover, 2015; Mayer et al., 2015). Recent studies have initiated the application of functional magnetic resonance imaging (fMRI), a non-invasive neuroimaging technique that leverages magnetic resonance imaging to measure the hemodynamic changes caused by neuronal activity (Chen and Glover, 2015; Mayer et al., 2015), to elucidate the neural substrates underlying figurative language comprehension in individuals with ASD (Harris et al., 2006).

Neuroimaging research on figurative language comprehension in individuals with ASD has predominantly concentrated on metaphor, irony and puns (Chouinard et al., 2017; Colich et al., 2012; Graves et al., 2022; Kana and Wadsworth, 2012). For example, Chouinard et al. (2017) employed a sentence decision task to assess the selection stage of metaphor comprehension in autistic individuals, which necessitates the suppression of unintended meaning to select the intended meaning. Their findings indicated that, compared to neurotypical controls (NCs), individuals with ASD recruited greater activation in the thalamus, middle temporal gyrus, and middle occipital gyrus. Conversely, Graves et al. (2022) observed that during figurative speech processing, the ASD group lacked activation in the posterior cingulate cortex and middle temporal gyrus (MTG) relative to NCs. While neuroimaging studies have consistently identified differences in brain activation between ASD and NCs during figurative language comprehension tasks, there is no consensus on specific abnormal brain activation patterns in ASD individuals due to variations in sample size, experimental designs, tasks, and analytical methods across studies. This inconsistency raises questions about the stability and consistency of brain activation patterns associated with figurative language understanding in individuals with ASD. Meta-analysis, an effective approach for amplifying sample size and statistical power (Sun et al., 2020), can synthesize findings from individual studies to pinpoint brain regions consistently activated across studies (Yu et al., 2022). Therefore, the present study conducted an activation likelihood estimation (ALE) meta-analysis of fMRI studies to examine the similarities and differences between ASD and NCs, aiming to uncover consistent brain activation patterns associated with figurative language comprehension in autistic individuals.

## 2 Materials and methods

### 2.1 Data sources and study selection

This study adhered to the Preferred Reporting Items for Systematic reviews and Meta-Analyses Statement (PRISMA; Page et al., 2021; refer to Supplementary Tables S1, S2), and was registered with the PROSPERO International Prospective Register of Systematic Reviews under the identifier CRD42023467185. The registration is available at http://www.crd.york.ac.uk/PROSPERO. A comprehensive and systematic search was conducted by two independent authors across Chinese and English databases, including PubMed, Embase, Web of Science, Chinese National Knowledge Infrastructure (CNKI), and Wanfang Data up to January 31st, 2025 to identify fMRI studies exploring neural similarities and differences in figurative language comprehension between individuals with ASD and NCs. The search employed various combinations of the following keywords in English: (‘ASD’ OR ‘autism’ OR ‘autism spectrum disorder’) AND (‘fMRI’ OR ‘functional magnetic resonance imaging’ OR ‘functional MRI’ OR ‘neuroimaging’) AND (‘metaphor’ OR ‘metaphoric’ OR ‘figurative’ OR ‘irony’ OR ‘ironic’ OR ‘pun’ OR ‘metonymy’ OR ‘metonymic’ OR ‘idiom’ OR ‘sarcasm’). Due to the limited number of fMRI studies on language processing in ASD published in Chinese journals (Yu et al., 2022), the search terms used for Chinese literature were slightly adjusted. Diagnostic terms such as “autism” and “ASD” were combined with methodological terms such as “fMRI,” “functional magnetic resonance imaging,” or “neuroimaging” and with terms related to figurative language comprehension tasks such as “metaphor,” “metonymy,” “pun,” “irony,” “sarcasm,” “idiom” or “figurative” in Chinese. The search was limited to sources in the Guide of the core Journal of China, Chinese Social Sciences Citation Index (CSSCI) and Chinese Science Citation Database (CSCD). Detailed search strategies for each database were provided in Supplementary Table S3. Additionally, the references of included studies and relevant review articles were also scrutinized to ensure the comprehensiveness in the search process.

For this meta-analysis, the inclusion criteria were as follows, studies must have been: (1) published journal articles; (2) included both ASD and NCs; (3) required participants to perform figurative language comprehension tasks during fMRI scan; (4) used whole-brain analysis to derive brain activation results; (5) reported peak activation coordinates in standard stereotactic coordinates, such as the Montreal Neurological Institute (MNI) coordinates or Talairach coordinates; (6) provided brain activation coordinates for NCs or ASD participants, or identified regions with greater activation in ASD compared to NCs (ASD > NCs) or vice versa (NCs > ASD) during figurative language comprehension tasks. Meanwhile, studies were excluded if they: (1) were non-empirical such as conference abstracts or review articles; (2) did not use fMRI technique; (3) used region-of-interest (ROI) or seed-based analysis instead of whole-brain analysis; (4) fail to report peak activation coordinates; (5) did not show significant difference in intra-group / inter-group comparison.

Following the screening process, two authors independently and rigorously assessed the quality and Risks of Bias (RoB) of each included study using a modified version of the Newcastle-Ottawa scale (NOS; mNOS), specifically tailored for fMRI studies by incorporating an assessment of statistical analysis (Costa et al., 2021; Gentili et al., 2019). The modified mNOS scores range from 0 to 11, with scores 0 to 3 indicating high risk, 4 to 7 indicating medium risk, and 8 to 11 indicating low risk of bias (Costa et al., 2021; Gentili et al., 2019). In cases of disagreement between the two authors, a discussion was held to reach a consensus on the final assessment.

### 2.2 Data extraction

Data extraction was performed independently by two authors, who collated the following details from each eligible study: (1) participant characteristics, including the number, gender, mean age, and ASD diagnostic method; (2) features of figurative language comprehension tasks, encompassing task types, stimulus language, conditions included in the tasks, and the behavioral results of both ASD and NCs; (3) four types of contrasts, namely, brain regions associated with figurative language comprehension in both ASD and NCs, respectively (defined as task-activated brain area minus baseline-activated brain area for both groups), regions with heightened activation in ASD individuals relative to NCs during figurative language processing (ASD > NCs), and regions with greater activation in NCs compared to ASD individuals (NCs > ASD; Tryfon et al., 2018). Meanwhile, the numbers of studies, included participants and reported foci for each contrast were also extracted.

### 2.3 Activation likelihood estimation meta-analysis

In this study, an ALE meta-analysis was performed in standard MNI space using Ginger ALE 3.0.2 software.^1^ Prior to the analysis, all Talairach coordinates of the reported foci were converted into MNI space using the icbm2tal conversion tool (Lancaster et al., 2007). To estimate the possibility of cross-experimental activation for each voxel under certain conditions, ALE meta-analysis calculated the modeled activation map (MA) for each experiment separately, namely the activation possibility of each voxel in the whole brain under certain conditions in each experiment. Specifically, in ALE meta-analysis, each foci was regarded as the center of a three-dimensional Gaussian distribution (Phan et al., 2021). The probability of each voxel being part of the brain region represented by each foci was calculated using three-dimensional Gaussian simulations, with the full width at half maxima (FWHM) being determined by the number of participants in each study (Eickhoff et al., 2009). Subsequently, the MA maps from each experiment were combined at voxel level to obtain the activation possibility of each voxel across the experiment (Hu et al., 2015). Ultimately, stable activation voxel clusters across experiments were obtained by carrying out significance testing and multiple comparison correction for the activation possibility of each voxel across the experiments (Eickhoff et al., 2012; Eickhoff et al., 2009).

The ALE meta-analyses were initially conducted separately for NCs and individuals with ASD. Cluster-level inference was applied to identify brain regions that were consistently activated during figurative language comprehension in both groups, using a cluster-level family-wise error (FWE) rate of 0.05, 1,000 permutations threshold and a *p* value threshold of 0.001 (Yu et al., 2022). This was followed by a pooled analysis to amalgamate foci from both groups into a single file, using the same parameters. Subsequently, a conjunction analysis was performed to reveal brain activation regions common to both NCs and ASD, along with contrast analyses to evaluate differences in brain activation between the two groups (NCs – ASD / ASD – NCs). These analyses were based on the generated ASD, NCs and pooled ALE maps, with a significance level set at *p* value = 0.05, *p* value Permutations = 1,000, and a Minimum Volume threshold of 0 mm^3^ (Eickhoff et al., 2012; Eickhoff et al., 2009). Furthermore, inter-group analyses were conducted to investigate consistent brain activation regions across studies where one group showed heightened activation than the other by using the reported coordinates of group differences (NCs > ASD and ASD > NCs). These analyses were conducted with a Cluster-level FWE of 0.05, 1,000 permutations threshold =, and a *p* value of 0.001 (Tryfon et al., 2018). Ultimately, the results of ALE meta-analyses were reported in standard MNI coordinates.

## 3 Results

### 3.1 Study selection

**Figure 1:**
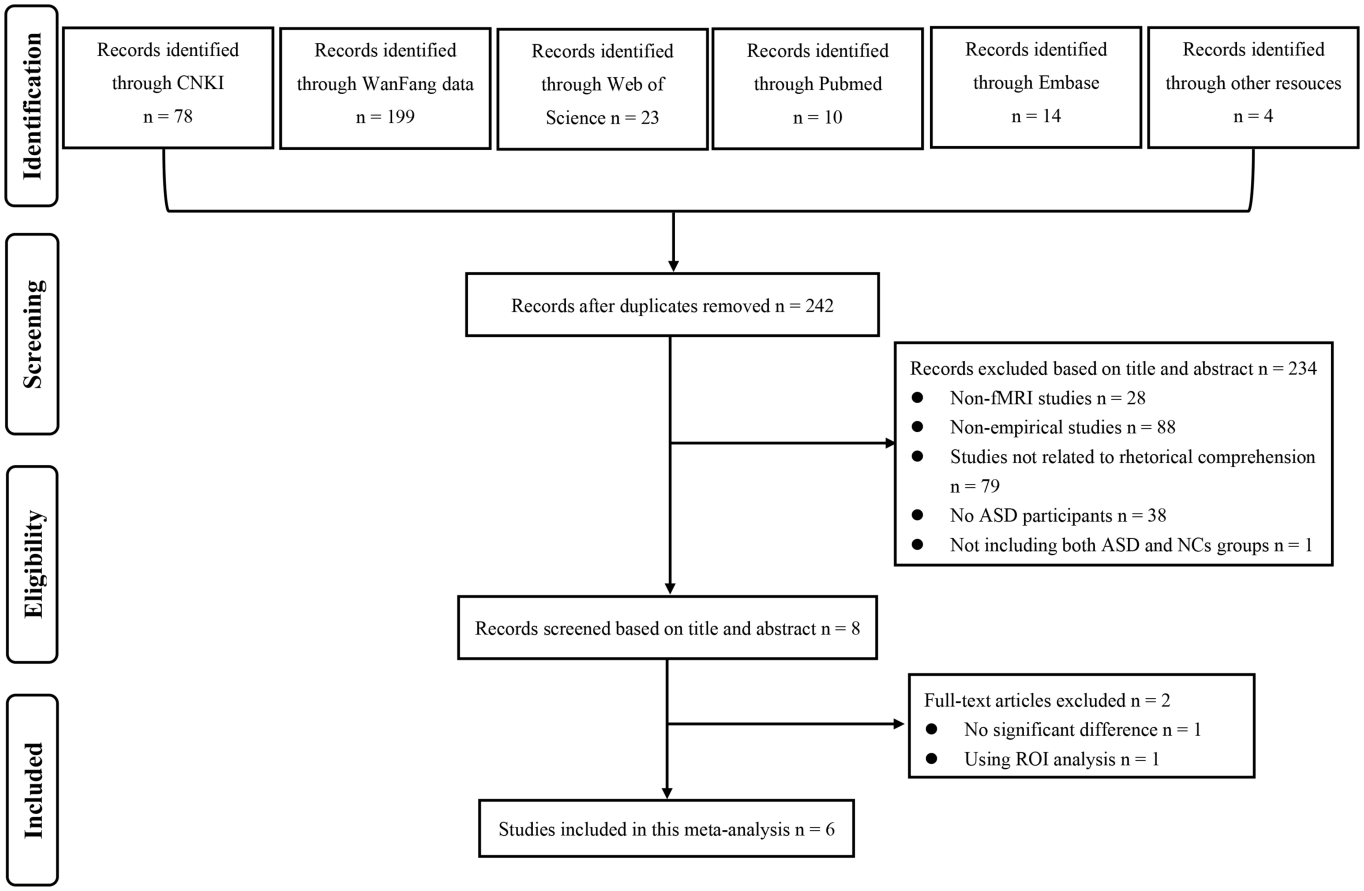
PRISMA flow chart depicting the selection process for included studies in this meta-analysis. CNKI, Chinese National Knowledge Infrastructure; fMRI, functional magnetic resonance imaging; ASD, autism spectrum disorder; NCs, neurotypical controls; ROI, region of interests.

After screening 328 studies sourced from five databases and related references, a stringent application of inclusion and exclusion criteria led to the exclusion of 86 studies due to duplication, 28 for not utilizing fMRI technology, 88 for being non-empirical (e.g., abstract, reviews), 79 for irrelevance to figurative language comprehension tasks, 38 for lacking ASD participants, and one for not comparing ASD and NCs. An additional study was excluded after full-text screening due to the absence of significant differences in figurative language comprehension between ASD and NCs, and another for employing ROI analysis. The excluded studies and rationales were detailed in Supplementary Table S4. Ultimately, six studies were included in the meta-analysis, as depicted in the PRISMA flow chart in Figure 1.

### 3.2 Characteristics of included studies

In this meta-analysis, encompassing 6 studies, there were 95 participants with ASD (mean ± SD age: 15.9939 ± 8.636 years) and 98 matched controls (mean ± SD age: 15.9904 ± 8.407 years; Chouinard et al., 2017; Colich et al., 2012; Kana and Wadsworth, 2012; Kim et al., 2018; Wang et al., 2006, 2007). In terms of ASD diagnosis, 3 studies utilized expert clinical assessments in accordance with DSM-IV, supplemented by ADOS and ADI criteria (Colich et al., 2012; Kim et al., 2018; Wang et al., 2007), 2 studies relied on ADI-R and ADOS-G (Kana and Wadsworth, 2012; Wang et al., 2006), and the remaining study employed ADOS-2 criteria (Chouinard et al., 2017). Table 1 presented the fundamental participant demographics included in this meta-analysis.

**Table 1:** Basic information of participants included in this meta-analysis.

Studies	Participants (F)		Mean age (SD)		ASD diagnostic methods	Stimulus language
	ASD	NCs	ASD	NCs		
Chouinard et al. (2017)	12 (3)	12 (4)	32.4 (10.8)	33.0 (10.1)	ADOS-2	English
Colich et al. (2012)	16 (2)	16 (2)	14.27 (2.5)	13.15 (2.18)	ADOS, ADI, expert clinical diagnosis based on DSM-IV	English
Kana and Wadsworth (2012)	16 (0)	16 (0)	20 (6.43)	21.6 (2.70)	ADI-R, ADOS-G	English
Kim et al. (2018)	15 (1)	18 (8)	9.66 (2.19)	10.47 (2.78)	ADI-R, ADOS, expert clinical diagnosis based on DSM-IV	Korean
Wang et al. (2006)	18 (0)	18 (0)	11.9 (2.8)	11.9 (2.3)	ADI-R, ADOS-G	English
Wang et al. (2007)	18 (0)	18 (0)	12.4 (2.9)	11.8 (1.9)	ADOS, ADI-R, Expert clinical diagnosis based on DSM-IV	English

F, female; ASD, autism spectrum disorder; NCs, neurotypical controls; SD, standard deviation; ADOS, Autism Diagnosis Observation Schedule; ADI, Autism Diagnostic Interview; DSM, The diagnostic and statistical manual of mental disorders.

Table 2 summarized the fundamental details of the six studies included in this meta-analysis. Regarding the language of experimental material, five studies used English stimuli (Chouinard et al., 2017; Colich et al., 2012; Kana and Wadsworth, 2012; Wang et al., 2006, 2007), while one study utilized Korean stimuli (Kim et al., 2018). The meta-analysis primarily encompassed three types of figurative language comprehension tasks: (1) participants judged the veracity of the literal meaning of sentence (Chouinard et al., 2017); (2) participants assessed whether the sentence meaning matched the scene depicted by a picture, namely, whether the speaker’s words in the situation presented by the picture really mean what he/she is saying (Colich et al., 2012; Kim et al., 2018; Wang et al., 2006, 2007); (3) participants engaged in reading and understand the meaning of sentences, including puns and control statements (Kana and Wadsworth, 2012).

**Table 2:** Summary of studies included in this meta-analysis.

Studies	Tasks	Behavioral results	Conditions	ASD	NCs	ASD>NCs	NCs>ASD
Chouinard et al. (2017)	Literal sentence meaning judgment	Both groups showed metaphorical interference effect.	Metaphor > scrambled metaphor	✓	✓	✓	
Colich et al. (2012)	Sentence meaning and scene matching judgment	There was no difference in accuracy between groups. Both groups showed longer RT for ironic remarks.	Ironic scenarios vs. resting-state baseline	✓	✓	✓	✓
Kana and Wadsworth (2012)	Sentence comprehension	No behavioral data was collected.	Pun comprehension vs. fixation			✓	
Kim et al. (2018)	Sentence and picture matching judgment	There were no significant differences in RT between NC and ASD children in both M and MM conditions. Participants with ASD showed significantly lower accuracy than NC only in the MM condition.	Metaphorical meaning vs. literal meaning				✓
Wang et al. (2006)	Sincere/ironic sentence meaning judgment	Event outcome + prosodic cues (sincere/sarcastic): the accuracy of NCs was higher than that of ASD group; prosodic cues only: there was no difference in accuracy between the two groups; Under both conditions, there was no difference in RT between the two groups.	Event outcome + prosodic cues (sincere/sarcastic)/ prosodic cues only vs. resting-state baseline	✓	✓	✓	
Wang et al. (2007)	Sentence meaning and scene matching judgment	There were no significant differences in RT and accuracy between groups.	Ironic vs. control scenarios	✓	✓	✓	✓

ASD, autism spectrum disorder; NCs, neurotypical controls; RT, response time; M, matched; MM, mismatched.

The mean kappa statistic, which measures the agreement between the two authors, was 0.81 ± 0.072 (Mean ± SD), with the values ranging from 0.4 to 1. According to the assessment criteria, three studies were classified as having an intermediate risk of bias, while the remaining three were classified as low risk of bias. The detailed quality and RoB assessments for the included studies were presented in the Supplementary material, specifically in Supplementary Tables S5, S6.

### 3.3 Results of ALE meta-analyses

**Figure 2:**
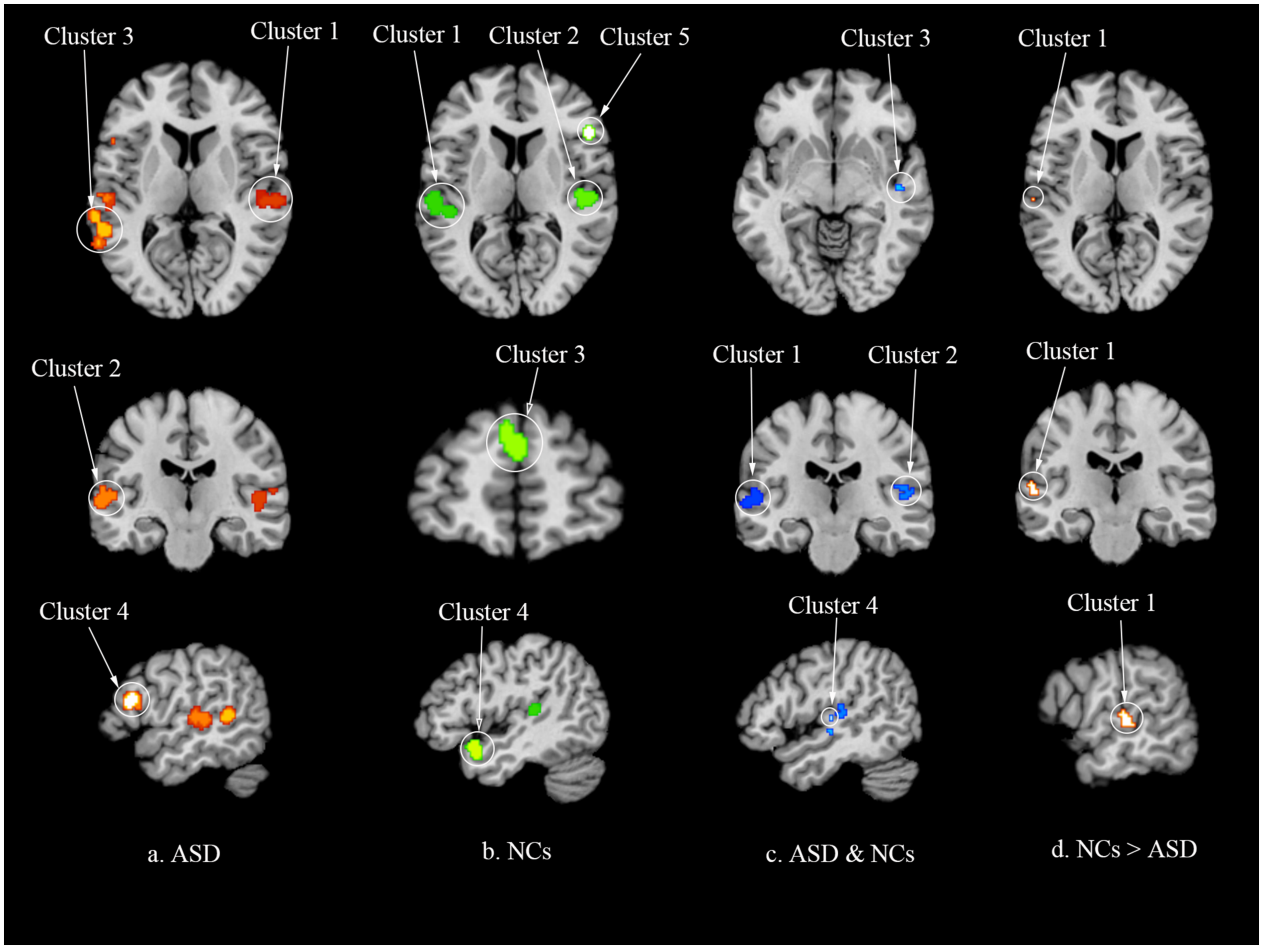
The results of ALE meta-analysis. **(a)** Activation clusters in individuals with autism spectrum disorder (ASD). **(b)** Activation clusters in neurotypical controls (NCs). **(c)** Overlapping activation clusters in ASD and NCs. **(d)** Different activation clusters between ASD and NCs. ALE, activation likelihood estimation; ASD, autism spectrum disorder; NCs, neurotypical controls.

For NCs, a total of 64 participants across four studies contributed 141 foci that formed five distinct clusters of activation (Tables 3, 4; Figure 2). The first and largest cluster was located in the left temporal lobe, comprising 61.4% of the STG, 28.7% of the middle temporal gyrus, and 9.9% of the transverse temporal gyrus (TTG). The second cluster was primarily identified in the right temporal lobe (63.5%) while a minor portion in the right sub-lobar region (36.5%), consisting of 46.1% of the STG, 35.9% of the insula (INS), 14.4% of the TTG and 3.6% of the sub-gyral. The third cluster was observed in the left frontal lobe, with 84.1% of the medial frontal gyrus and 15.9% of the superior frontal gyrus (SFG). The fourth cluster was discovered in the left brain, consisting of 79.6% of the STG and 20.4% of the inferior frontal gyrus (IFG). The fifth cluster was located in the right frontal lobe, consisting of 83.3% of the IFG and 16.7% of the precentral gyrus (PreCG).

**Table 3:** ALE contrasts specifying number of studies, foci and participants.

Contrast	Studies	Foci	Participants
ASD	4	122	64
NCs	4	141	64
ASD > NCs	5	20	80
NCs > ASD	3	35	49

ALE, Activation Likelihood Estimation; ASD, autism spectrum disorder; NCs, neurotypical controls.

**Table 4:** ALE clusters of activation during the figurative language speech comprehension processing.

Conditions	Brain region	BA	MNI coordinates	Cluster size (mm^3^)	Maximum ALE value	Studies contributing to cluster
			(x,y,z)			
ASD	Right Superior Temporal Gyrus	41	(53.1,−22.1,4.6)	2008	0.0163	2 foci from Colich et al. (2012) 7 foci from Wang et al. (2006) 3 foci from Wang et al. (2007)
	Left Superior Temporal Gyrus	22	(−58.1,−23.2,1.8)	1,552	0.0166	1 foci from Colich et al. (2012) 5 foci from Wang et al. (2006) 2 foci from Wang et al. (2007)
	Left Middle Temporal Gyrus	22	(−60.2,−44.5,7.2)	1,472	0.0162	1 foci from Colich et al. (2012) 3 foci from Wang et al. (2006) 3 foci from Wang et al. (2007)
	Left Inferior Frontal Gyrus	45	(−53.3,21.2,15.1)	904	0.0177	2 foci from Colich et al. (2012) 2 foci from Wang et al. (2006) 1 foci from Wang et al. (2007)
NCs	Left Superior Temporal Gyrus	41	(−57,−25.3,4.3)	2,464	0.0223	1 foci from Colich et al. (2012) 6 foci from Wang et al. (2006) 7 foci from Wang et al. (2007)
	Right Superior Temporal Gyrus	13	(48.6,−18.1,3.6)	2,392	0.0146	2 foci from Colich et al. (2012) 8 foci from Wang et al. (2006) 7 foci from Wang et al. (2007)
	Left Medial Frontal Gyrus	9	(−0.8,53.3,25)	1,384	0.0185	1 foci from Colich et al. (2012) 2 foci from Wang et al. (2006) 4 foci from Wang et al. (2007)
	Left Superior Temporal Gyrus	38	(−46.5,12.3,−19.3)	856	0.0156	1 foci from Colich et al. (2012) 2 foci from Wang et al. (2006) 2 foci from Wang et al. (2007)
	Right Inferior Frontal Gyrus	45	(51.1,23.9,6.9)	760	0.0154	1 foci from Colich et al. (2012) 2 foci from Wang et al. (2006) 2 foci from Wang et al. (2007)
NCs & ASD	Left Superior Temporal Gyrus	22	(−57.6,−22.8,3.1)	1,016	0.0147	1 foci from Colich et al. (2012) 3 foci from Wang et al. (2006) 4 foci from Wang et al. (2007) 1 foci from Colich et al. (2012) 3 foci from Wang et al. (2006) 1 foci from Wang et al. (2007)
	Right Superior Temporal Gyrus	41	(50.3,−23.1,8.5)	400	0.0118	2 foci from Wang et al. (2006) 2 foci from Wang et al. (2006)
	Right Insula	13	(48.2,−15,−7.3)	64	0.0101	1 foci from Colich et al. (2012)
	Right Insula	13	(50,−16,3)	16	0.0088	
ASD − NCs	none					
NCs − ASD	none					
ASD > NCs	none					
NCs > ASD	Left Superior Temporal Gyrus	22	(−58.2,−25.3,1.3)	560	0.0109	1 foci from Colich et al. (2012) 3 foci from Wang et al. (2007)

ALE, Activation Likelihood Estimation; BA, Brodmann Area; MNI, Montreal Neurological Institute; ASD, autism spectrum disorder; NCs, neurotypical controls.

In the ASD group, four studies encompassing 64 participants reported 122 foci that contributed to the formation of four activation clusters (Tables 3, 4; Figure 2). The first and largest cluster was predominantly found in the right temporal lobe (71.3%), with a minor portion in the right sub-lobar region (28.7%), comprising 50.8% of the STG, 30.3% of the INS, and 18.9% of the TTG. The second cluster was observed in the left temporal lobe, consisting of 77.6% of the STG, 17.1% of the MTG, and 5.3% of the TTG. The third cluster was also observed in the left temporal lobe, made up of 69.1% of the MTG and 30.9% of the STG. The fourth cluster was discovered in the left IFG.

The conjunction analysis identified four activation clusters across NCs and ASD individuals (Table 4; Figure 2). The first cluster was located in the left temporal lobe, comprising 88.9% of the STG and 11.1% of the TTG. The second cluster was detected in the right temporal lobe, with an equal distribution of 50% in the STG and 50% in the TTG. The third cluster spanned the right inferior lobe (85.7%) and right temporal lobe (14.3%), consisting of the INS (85.7%) and the STG (14.3%). The fourth cluster was centered in the right INS. In the subtraction analysis, no significant activation clusters were observed in either the ASD–NCs or NCs–ASD contrasts.

Three studies reported heightened brain activation in NCs compared to ASD participants, with a total of 35 foci identified (Table 3). Following the ALE meta-analysis for group differences (NCs > ASD), a single cluster emerged in the left temporal lobe, consisting of 66.7% of the STG and 33.3% of the MTG (Table 4; Figure 2). Conversely, five studies indicated increased brain activation in ASD participants compared to NCs, with 20 foci reported (Table 3). However, the ALE analysis for this contrast did not yield any significant clusters (Table 4; Figure 2).

## 4 Discussion

This study aimed to uncover consistent brain activation patterns associated with figurative language comprehension in individuals with ASD by conducting an ALE meta-analysis of six fMRI studies. The principal findings were summarized as follows: (1) in NCs, figurative language comprehension consistently activated five clusters, primarily located in the bilateral STG, left medial frontal gyrus and right IFG; (2) in the ASD group, four clusters were consistently activated during the tasks, mainly comprising the bilateral STG, left MTG, and left IFG; (3) the conjunction analysis revealed four shared clusters between ASD and NCs, predominantly encompassing the bilateral STG and the right insula. However, no significant activation clusters were found in either the ASD–NCs or NCs–ASD contrasts; (4) in the NCs > ASD contrast, a single cluster, concentrated in the left STG, was identified.

The ALE meta-analysis for NCs revealed five significant clusters, with the most prominent one located in the left STG, MTG, and TTG. The STG, which includes the primary auditory cortex and Wernicke’s area, serves as the auditory speech center. It is critical for auditory processing, auditory short-term memory, and language processing, playing a central role in integrating diverse information to derive appropriate meanings (Boddaert and Zilbovicius, 2002; Jou et al., 2010). The involvement of the STG also extends to theory of mind (ToM; Baron-Cohen et al., 1999; Cheng, 2023) and social perception (Jou et al., 2010), highlighting its multifaceted contribution to cognitive functions. In the context of figurative language processing, the activation of the STG may reflect its role in auditory processing, semantic integration, and reasoning, all of which are essential for understanding the underlying meanings of figurative expressions (Happé, 1993).

The left MTG is a key part of the ventral pathway, which links sounds to meaning (Hickok and Poeppel, 2007), and is involved in the intelligibility of sentences and meaningful phonics (Davis and Johnsrude, 2003; Price, 2010). Neuroimaging studies on the semantic processing of polysemous words have consistently reported activation in both the MTG and STG (Caplan and Dapretto, 2001; Rodd et al., 2005). This suggests that these regions are strongly associated with the activation and competition of multiple word meanings in figurative language comprehension. The TTG, also known as Heschl’s gyrus, contains the primary auditory cortex (Gaser et al., 2004) and is engaged in the initial stages of speech processing, including the analysis of frequency, duration, and intensity (Wang et al., 2019). The activation of the TTG in this meta-analysis is likely attributed to the dominance of auditory stimuli in the figurative language comprehension tasks of the included studies, thereby activating early speech processing mechanisms within the primary auditory cortex.

The second cluster, comprising the right STG, INS, TTG, and sub-gyral, plays a crucial role in figurative language comprehension. The INS, located adjacent to Broca’s area and Wernicke’s area, is integral to the cognitive-emotional circuit involved in sentence processing. It coordinates the language regions of the frontal and temporal lobes (Kurth et al., 2010; Schmidt and Seger, 2009), facilitating the integration required for meaning activation and selection. In the context of figurative language comprehension, this may involve enhanced temporal-lobe engagement for meaning activation, coupled with frontal-lobe involvement for meaning selection. The INS also plays a pivotal role in social communication, particularly in the perception, recognition, and understanding of emotional expressions (Uddin et al., 2017; Zhong et al., 2023). Since figurative language, such as irony, can vividly convey the speaker’s emotions and attitudes, the observed activation of the right INS in NCs may reflect this emotional aspect of figurative language. This finding is consistent with previous studies that reported bilateral INS activation in healthy participants during metaphorical comprehension tasks (Ahrens et al., 2007; Kuperberg et al., 2000; Mashal et al., 2007). Additionally, a recent large-scale study has highlighted the sub-gyral as a critical region in human working memory, involved in the temporary storage and manipulation of information (Sadeghi et al., 2021; Yao et al., 2023). The comprehension of figurative language requires reasoning about non-literal meanings and relies on cognitive skills associated with working memory, including the temporary storage and manipulation of information. This suggests that the activation of the sub-gyral in our meta-analysis is likely due to the cognitive demands posed by figurative language processing.

The third cluster identified in our study was located in the left medial and superior frontal gyrus. The medial frontal gyrus, encompassing the pre-supplementary motor area and supplementary motor area (Phan et al., 2021), is associated with a range of general cognitive functions essential for task completion (Yu et al., 2022), such as information gathering (Erdler et al., 2000) and decision-making (Nachev et al., 2007). During figurative language comprehension tasks, the activation of the left medial frontal gyrus suggests a role in aggregating information from various brain areas, thereby aiding in semantic selection during the interpretation of figurative expressions. The SFG is particularly implicated in the working memory demands of task execution (Petrides, 2005), with a focus on execution and attention (Li et al., 2013). It also plays a role in syntactic processing and the understanding of polysemous words (Newman and Twieg, 2001). Consistent with our findings, previous research has indicated that the left SFG was activated in context where word meaning is contingent upon semantic context (Price, 2012; Scott et al., 2003), highlighting its importance in the dynamic interpretation of language.

The fourth cluster was in the left STG and IFG. Activation of the left IFG is associated with semantic retrieval and selection tasks (Lauro et al., 2008; Oh et al., 2014), and it may serve as a central monitoring hub for semantic processing. This region is sensitive to the depth of semantic processing, reflecting the higher-level processing demands of deep semantic reasoning in figurative language comprehension. It also plays a role in further searching for semantic information related to word meanings, thereby integrating the semantic meaning of figurative expressions (Li et al., 2016). The fifth activation cluster, situated in the right IFG and PreCG, is noteworthy for its involvement in speech processing. The PreCG, a significant component of the motor language area (Rouzi et al., 2022), is activated when participants are exposed to speech stimuli during figurative language tasks (Ripamonti et al., 2018). The activation supports the theory of embodied cognition in neuroscience and psychology (Ye, 2010), suggesting that both real and imagined body movements can prime the comprehension of metaphorical verbal phrases. Matched body movements, in particular, have been shown to enhance understanding of such phrases. In summary, the ALE meta-analysis revealed that the brain regions activated in the five clusters within NCs are closely intimately connected with the processing of figurative language comprehension. These findings underscore the complex interplay between semantic retrieval, semantic monitoring, and embodied cognition in the neural underpinnings of understanding figurative language.

The ALE meta-analysis of the ASD group revealed four clusters, with the most significant located in the right STG, INS and TTG. Additional clusters were identified in the left STG, MTG and TTG, as well as in the left MTG and STG, and finally in the left IFG. This pattern of activation in both ASD and NCs during figurative language comprehension tasks suggests that the neural underpinnings of figurative language comprehension engage a broad range of bilateral distributed systems, aligning with previous research (Bottini et al., 1994). The brain regions consistently activated across studies in ASD participants were also observed in NCs, highlighting their relevance to speech processing, information integration, deep semantic processing, and psychological inference. These regions are integral to the core neural network responsible for language processing (Herringshaw et al., 2016). The overlap in activation patterns between ASD and NCs groups underscores the shared neural mechanisms involved in figurative language comprehension, despite the potential for variability in processing depth and efficiency within the ASD group.

The conjunction analysis revealed four common clusters in both ASD and NCs during the processing the figurative language. The first cluster was located in the left STG and TTG, while the second was in the right STG and TTG. The third cluster was identified in right INS and STG, and the fourth in the right INS. These regions are crucial for auditory processing, semantic activation and competition, suppression of literal meaning, and reasoning of non-literal meaning. The conjunction analysis results underscore the stability of activation in these brain regions across studies for both ASD individuals and controls, suggesting a consistent neural substrate for figurative language comprehension. Subtraction analyses did not identify any activation clusters unique to either ASD or NCs. This absence of differential activation indicates that there were no additional brain regions activated in the ASD group compared to the NCs, and vice versa. The findings suggest that the core neural mechanisms engaged during figurative language comprehension are similar between ASD individuals and neurotypical controls, with no significant group-specific activation patterns detected.

The inter-group difference analysis revealed distinct patterns of brain activation between ASD and NCs during figurative language processing. The ALE meta-analysis comparing ASD to NCs did not yield any consistent activation clusters, suggesting no significant hyperactivity in ASD. Conversely, the ALE meta-analysis for NCs over ASD identified one cluster in the left STG and MTG, indicating hypoactivity in these regions for ASD individuals during figurative language comprehension. The hypoactivity observed in ASD participants in these regions can be explained from two interrelated aspects: semantic processing efficiency and social-cognitive integration.

First, the posterior left temporal lobe, encompassing the STG and MTG, serves as a key node in the ventral stream of the auditory-language dual-stream model, primarily responsible for mapping auditory signals to semantic concepts and supporting lexical access and semantic integration (Hickok and Poeppel, 2007; Price, 2012). The observed hypoactivation in this region may reflect reduced efficiency in information transfer and cooperative activation within the semantic network in ASD (Herringshaw et al., 2016). In the context of figurative language comprehension, this inefficiency could manifest as: difficulty in rapidly suppressing the dominant, automatically activated literal meaning, coupled with insufficient recruitment of neural resources when integrating disparate semantic features to construct a non-literal interpretation (Chouinard and Cummine, 2016). This aligns with the Weak Central Coherence theory in ASD research, which posits that individuals with ASD exhibit a bias toward local detail processing and experience difficulties in integrating information to derive global meaning or higher-order concepts (Frith, 1989).

Second, the comprehension of figurative language requires individuals to not only grasp the meanings of words but also to infer the speaker’s intentions, attitudes and emotions, which is a socio-linguistic interactive process. The left temporal lobe, particularly the MTG and STG, is not only a classical language region but is also considered a crucial component of the “social brain” network, involved in theory of mind and social perception (Arutiunian et al., 2023; Baron-Cohen et al., 1999). Therefore, the hypoactivation in the left STG/MTG may signify more than an inefficiency in lower-level semantic processing; it could be a neural marker of a higher-order functional challenge—namely, the difficulty individuals with ASD face in integrating linguistic cues with social intent. When processing figurative language such as irony or metaphor, which heavily relies on social context, the insufficient engagement of this region might prevent the crucial transition from “literal meaning” to “communicative meaning,” resulting in a literal interpretation (Kalandadze et al., 2018).

In summary, the hypoactivation in the left temporal lobe provides a unified neurobiological explanatory framework for figurative language comprehension deficits in ASD: it may reflect both an efficiency deficit in rapid and flexible information processing within the semantic network and a functional weakness in higher-order integration at the interface of language processing and social cognition. This offers novel evidence for connecting the language-specific deficits in ASD with their broader social communication impairments at the neural level.

## 5 Conclusion

This meta-analysis, based on an ALE meta-analysis of six fMRI studies, examined the consistent patterns of brain activation in figurative language comprehension among individuals with ASD compared to controls. The findings revealed that while ASD engaged the bilateral STG, TTG and right INS—brain regions typically associated with figurative language comprehension—the activation patterns in these areas were stable when compared to NCs. However, hypoactivation was observed in the left STG and MTG, suggesting a potential deficit in ASD individuals’ ability to suppress the literal meaning of figurative language and infer its non-literal meaning.

This meta-analysis has shed light on the atypical neural processing involved in figurative language comprehension among individuals with ASD. Nonetheless, the study is not without its limitations. The primary constraint is the inconsistency in the activation coordinates reported across studies, with some providing data for both intragroup and intergroup comparisons, while others only for one. This variability means that the datasets for these analyses are not entirely congruent, potentially impacting the meta-analysis’s conclusions. Secondly, although the rigorous inclusion criteria resulted in a modest sample of six studies, the consistency of our core findings across these studies underscores their robustness. Future meta-analyses would benefit from the growing number of high-quality fMRI studies in this domain, which will allow for subgroup analyses (e.g., by age, language, or type of figurative language) and enhance generalizability. Furthermore, the reliance on published activation coordinates rather than the original statistical brain maps could introduce inaccuracies into the analysis (Jiang et al., 2017). The use of varying statistical thresholds across studies also poses a challenge, as these thresholds may affect the outcomes, by extension, the meta-analysis results. A significant limitation is the focus on high-functioning ASD individuals, with a notable absence of younger children and a disproportionate representation of female participants. This demographic bias limits the generalizability of the findings. Therefore, future studies should aim to include more diverse and representative samples to enhance the application of the findings. This would be particularly beneficial for informing early diagnosis, clinical intervention and educational strategies.

## Data Availability

The original contributions presented in the study are included in the article/Supplementary material, further inquiries can be directed to the corresponding authors.

## Footnotes

1
http://brainmap.org/ale/
