# Supplementary material for: Brain activation patterns of figurative language comprehension in individuals with autism spectrum disorder: an activation likelihood estimation meta-analysis
Source: Front Neurosci. 2026 Jan 30;20:1717020. doi: 10.3389/fnins.2026.1717020 (PMC12901445; doi:10.3389/fnins.2026.1717020)
Supplement: Supplementary file 1 [file Data_Sheet_1.docx]

**Supplementary Materials**

**Table S1** PRISMA 2020 checklist

| **Section and Topic** | **Item #** | **Checklist item** | **Location where item is reported** |
| --- | --- | --- | --- |
| **TITLE** | | |  |
| Title | 1 | Identify the report as a systematic review. | Title |
| **ABSTRACT** | | |  |
| Abstract | 2 | See the PRISMA 2020 for Abstracts checklist. | Abstract |
| **INTRODUCTION** | | |  |
| Rationale | 3 | Describe the rationale for the review in the context of existing knowledge. | Introduction |
| Objectives | 4 | Provide an explicit statement of the objective(s) or question(s) the review addresses. | Introduction |
| **METHODS** | | |  |
| Eligibility criteria | 5 | Specify the inclusion and exclusion criteria for the review and how studies were grouped for the syntheses. | Section 2.1 |
| Information sources | 6 | Specify all databases, registers, websites, organisations, reference lists and other sources searched or consulted to identify studies. Specify the date when each source was last searched or consulted. | Section 2.1 |
| Search strategy | 7 | Present the full search strategies for all databases, registers and websites, including any filters and limits used. | Section 2.1 |
| Selection process | 8 | Specify the methods used to decide whether a study met the inclusion criteria of the review, including how many reviewers screened each record and each report retrieved, whether they worked independently, and if applicable, details of automation tools used in the process. | Section 2.1 & 2.2 |
| Data collection process | 9 | Specify the methods used to collect data from reports, including how many reviewers collected data from each report, whether they worked independently, any processes for obtaining or confirming data from study investigators, and if applicable, details of automation tools used in the process. | Section 2.2 |
| Data items | 10a | List and define all outcomes for which data were sought. Specify whether all results that were compatible with each outcome domain in each study were sought (e.g. for all measures, time points, analyses), and if not, the methods used to decide which results to collect. | Section 2.1 |
|  | 10b | List and define all other variables for which data were sought (e.g. participant and intervention characteristics, funding sources). Describe any assumptions made about any missing or unclear information. | Section 2.2 |
| Study risk of bias assessment | 11 | Specify the methods used to assess risk of bias in the included studies, including details of the tool(s) used, how many reviewers assessed each study and whether they worked independently, and if applicable, details of automation tools used in the process. | Section 2.1 |
| Effect measures | 12 | Specify for each outcome the effect measure(s) (e.g. risk ratio, mean difference) used in the synthesis or presentation of results. | Section 2.2 |
| Synthesis methods | 13a | Describe the processes used to decide which studies were eligible for each synthesis (e.g. tabulating the study intervention characteristics and comparing against the planned groups for each synthesis (item #5)). | Section 2.2 |
|  | 13b | Describe any methods required to prepare the data for presentation or synthesis, such as handling of missing summary statistics, or data conversions. | Section 2.2 |
|  | 13c | Describe any methods used to tabulate or visually display results of individual studies and syntheses. | Section 2.2 |
|  | 13d | Describe any methods used to synthesize results and provide a rationale for the choice(s). If meta-analysis was performed, describe the model(s), method(s) to identify the presence and extent of statistical heterogeneity, and software package(s) used. | Section 2.2 |
|  | 13e | Describe any methods used to explore possible causes of heterogeneity among study results (e.g. subgroup analysis, meta-regression). | Section 2.2 |
|  | 13f | Describe any sensitivity analyses conducted to assess robustness of the synthesized results. | Section 2.2 |
| Reporting bias assessment | 14 | Describe any methods used to assess risk of bias due to missing results in a synthesis (arising from reporting biases). | Section 2.2 |
| Certainty assessment | 15 | Describe any methods used to assess certainty (or confidence) in the body of evidence for an outcome. | Section 2.2 |
| **RESULTS** | | |  |
| Study selection | 16a | Describe the results of the search and selection process, from the number of records identified in the search to the number of studies included in the review, ideally using a flow diagram. | Figure1 |
|  | 16b | Cite studies that might appear to meet the inclusion criteria, but which were excluded, and explain why they were excluded. | Figure1 |
| Study characteristics | 17 | Cite each included study and present its characteristics. | Section 3.1 |
| Risk of bias in studies | 18 | Present assessments of risk of bias for each included study. | Section 3.1 |
| Results of individual studies | 19 | For all outcomes, present, for each study: (a) summary statistics for each group (where appropriate) and (b) an effect estimate and its precision (e.g. confidence/credible interval), ideally using structured tables or plots. | Table2 |
| Results of syntheses | 20a | For each synthesis, briefly summarise the characteristics and risk of bias among contributing studies. | Section 3.1 & 3.2 |
|  | 20b | Present results of all statistical syntheses conducted. If meta-analysis was done, present for each the summary estimate and its precision (e.g. confidence/credible interval) and measures of statistical heterogeneity. If comparing groups, describe the direction of the effect. | Section 3.1 & 3.2 |
|  | 20c | Present results of all investigations of possible causes of heterogeneity among study results. | Section 3.1 & 3.2 |
|  | 20d | Present results of all sensitivity analyses conducted to assess the robustness of the synthesized results. | Section 3.1 & 3.2 |
| Reporting biases | 21 | Present assessments of risk of bias due to missing results (arising from reporting biases) for each synthesis assessed. | Section 3.1 & 3.2 |
| Certainty of evidence | 22 | Present assessments of certainty (or confidence) in the body of evidence for each outcome assessed. | Section 3.1 & 3.2 |
| **DISCUSSION** | | |  |
| Discussion | 23a | Provide a general interpretation of the results in the context of other evidence. | Discussion |
|  | 23b | Discuss any limitations of the evidence included in the review. | Conclusion |
|  | 23c | Discuss any limitations of the review processes used. | Conclusion |
|  | 23d | Discuss implications of the results for practice, policy, and future research. | Conclusion |
| **OTHER INFORMATION** | | |  |
| Registration and protocol | 24a | Provide registration information for the review, including register name and registration number, or state that the review was not registered. | Section 2.1 |
|  | 24b | Indicate where the review protocol can be accessed, or state that a protocol was not prepared. | Section 2.1 |
|  | 24c | Describe and explain any amendments to information provided at registration or in the protocol. | Section 2.1 |
| Support | 25 | Describe sources of financial or non-financial support for the review, and the role of the funders or sponsors in the review. | Funding |
| Competing interests | 26 | Declare any competing interests of review authors. | Declarations of Interest |
| Availability of data, code and other materials | 27 | Report which of the following are publicly available and where they can be found: template data collection forms; data extracted from included studies; data used for all analyses; analytic code; any other materials used in the review. | Data availability statement |

From: Page MJ, McKenzie JE, Bossuyt PM, Boutron I, Hoffmann TC, Mulrow CD, et al. The PRISMA 2020 statement: an updated guideline for reporting systematic reviews. BMJ 2021;372:n71. doi: 10.1136/bmj.n71 For more information, visit: <http://www.prisma-statement.org/>

**Table S2** PRIMSA Abstract Checklist

| **Topic** | **No.** | **Item** | **Reported?** |
| --- | --- | --- | --- |
| **TITLE** |  |  |  |
| **Title** | 1 | Identify this report as a systematic review. | Yes |
| **BACKGROUND** |  |  |  |
| **Objectives** | 2 | Provide an explicit statement of the main objective(s) or question(s) the review addresses. | Yes |
| **METHODS** |  |  |  |
| **Eligibility criteria** | 3 | Specify the inclusion and exclusion criteria for the review. | Yes |
| **Information sources** | 4 | Specify the information sources (e.g. databases, registers) used to identify studies and the date when each was last searched. | Yes |
| **Risk of bias** | 5 | Specify the methods used to assess risk of bias in the included studies. | Yes |
| **Synthesis of results** | 6 | Specify the methods used to present and synthesize results. | Yes |
| **RESULTS** |  |  |  |
| **Included studies** | 7 | Give the total number of included studies and participants and summarise relevant characteristics of studies. | Yes |
| **Synthesis of results** | 8 | Present results for main outcomes, preferably indicating the number of included studies and participants for each. If meta-analysis was done, report the summary estimate and confidence/credible interval. If comparing groups, indicate the direction of the effect (i.e. which group is favoured). | Yes |
| **DISCUSSION** |  |  |  |
| **Limitations of evidence** | 9 | Provide a brief summary of the limitations of the evidence included in the review (e.g. study risk of bias, inconsistency and imprecision). | Yes |
| **Interpretation** | 10 | Provide a general interpretation of the results and important implications. | Yes |
| **OTHER** |  |  |  |
| **Funding** | 11 | Specify the primary source of funding for the review. | Yes |
| **Registration** | 12 | Provide the register name and registration number. | Yes |

From: Page MJ, McKenzie JE, Bossuyt PM, Boutron I, Hoffmann TC, Mulrow CD, et al. The PRISMA 2020 statement: an updated guideline for reporting systematic reviews. MetaArXiv. 2020, September 14. DOI: 10.31222/osf.io/v7gm2. For more information, visit: [www.prisma-statement.org](http://www.prisma-statement.org)

**Table S3** Search strategies and results for each database

| **Database** | **Keywords** | **Results** |
| --- | --- | --- |
| **CNKI** | “自闭症/孤独症”(topic) AND “fMRI/功能磁共振(成像)/神经影像”(topic) 或 “自闭症/孤独症”(topic) AND “比喻/隐喻/转喻/反语/双关/讽刺”(topic)  (北大核心+CSSCI+SCI+CSCD) | 78 |
| **Wanfang data** | “自闭症/孤独症”(topic) AND “fMRI/功能磁共振(成像)/神经影像”(topic) 或 “自闭症/孤独症”(topic) AND “比喻/隐喻/转喻/反语/双关/讽刺”(topic)  (北大核心+CSSCI+CSCD+CSTPCD+SCI) | 199 |
| **Pubmed** | (("autism" OR "ASD" OR "autism spectrum disorder") AND ("functional magnetic resonance imaging" OR "fMRI" OR "functional MRI" OR "neuroimaging")) AND ("metaphor" OR "metaphoric" OR "figurative" OR "irony" OR "ironic" OR "pun" OR "metonymy" OR "metonymic" OR 'idiom' OR 'sarcasm') | 10 |
| **Web of Science** | ((TS=("ASD" OR "autism" OR "autism spectrum disorder")) AND TS=("functional magnetic resonance imaging" OR "fMRI" OR "functional MRI" OR "neuroimaging")) AND TS=("metaphor" OR "metaphoric" OR "figurative" OR "irony" OR "ironic" OR "pun" OR "metonymy" OR "metonymic" OR "sarcasm" OR "idiom") | 23 |
| **Embase** | ('asd'/exp OR 'asd' OR 'autism'/exp OR 'autism' OR 'autism spectrum disorder'/exp OR 'autism spectrum disorder') AND ('fmri'/exp OR 'fmri' OR 'functional magnetic resonance imaging'/exp OR 'functional magnetic resonance imaging' OR 'functional mri'/exp OR 'functional mri' OR 'neuroimaging'/exp OR 'neuroimaging') AND ('metaphor' OR 'figurative' OR 'irony' OR 'pun' OR 'metonymy' OR 'ironic' OR 'metaphoric' OR 'idiom' OR 'sarcasm' OR 'metonymic') | 14 |

**Table S4** Lists of excluded studies and reasons for exclusion

| **Overlapping studies** |
| --- |
| (Chen & Yang, 2014; Chen et al., 2023; Chen et al., 2010; Cheng et al., 2018; Cheng et al., 2016; Cheng & Liu, 2017; Chouinard et al., 2017; Colich et al., 2012; Cui, 2023; Dan et al., 2020; Deschrijver & Palmer, 2020; Graves et al., 2022; Gu et al., 2023; Guo et al., 2021; Herringshaw et al., 2016; Hu et al., 2021; Hu & Huang, 2014; Huang et al., 2017; Ibrahim et al., 2021; Ji et al., 2006; Jin & Liang, 2022; Kana & Wadsworth, 2012; Li, Hu, Liu, Li, et al., 2017; Li, Hu, Liu, Wang, et al., 2017; Li et al., 2016; Li et al., 2023; S. Li et al., 2015; Li, 2009; X. Li et al., 2015; Li & Liu, 2010; Li, 2014a, 2014b; Li, Ao, et al., 2022; Li, Zhang, et al., 2022; Y. Li et al., 2015; Liu et al., 2020; Liu & Miao, 2023; Liu et al., 2023; Liu et al., 2014; Lu et al., 2018; Lu et al., 2022; Lu et al., 2023; Lv et al., 2015; Ma et al., 2023; Peng et al., 2017; Shou et al., 2013; Simon et al., 2014; Song et al., 2021; Su et al., 2020; Wang et al., 2006, 2007; Wang et al., 2017; Wang et al., 2021; Wang & Zou, 2018; Wei & Yang, 2023; Williams et al., 2013; Xu & Cao, 2021; Yao et al., 2020; Ye & Gao, 2008; Yu et al., 2022; Zhang et al., 2020; Zhang, 2019; Zhang et al., 2015; Zhang et al., 2011; Zhang & Wang, 2021; Zhang & Zhang, 2007; Zhang et al., 2019; Zhao et al., 2021; Zhao & Jin, 2014; Zhong et al., 2023; Zhou & Li, 2007; Zhou et al., 2021) |
| **Not journal article** |
| (Levinson, 2021) |
| **Non-empirical studies** |
| (An et al., 2019; author, 2019; Bai et al., 2021; Cao & Fang, 2008; Chai et al., 2022; Chen & Chen, 2013; Chen et al., 2023; Chen & Jing, 2021; Chen et al., 2010; Chen & Lv, 2008; Cheng, 2023; Cheng et al., 2010; Chu et al., 2023; Cui et al., 2020; Di & Rao, 2007; Dong et al., 2020; Du et al., 2012; Fan et al., 2021; Fan et al., 2015; Fang et al., 2020; Fang et al., 2010; Fu & Ke, 2021; Gan et al., 2022; Gu et al., 2023; Herringshaw et al., 2016; Hu & Ye, 2005; Hu et al., 2021; Hu & Huang, 2014; Huang et al., 2003; Jia et al., 2020; Jing, 2017; Li et al., 2016; Li et al., 2012; S. Li et al., 2015; Li, 2009; X. Li et al., 2015; Li & Liu, 2010; Li & Zhou, 2006; Liang et al., 2014; Liu, 2019; Liu et al., 2021; Liu et al., 2020; Lu et al., 2017; Lu et al., 2022; Luo, 2011; Ma et al., 2016; Ma et al., 2023; Niu et al., 2023; Pan et al., 2022; Pan et al., 2020; Qu, 2016; She et al., 2022; Shou et al., 2013; Shou & Zhang, 2013; Su et al., 2014; Sun & Zhou, 2005; Sun, 2011; Tu & Xie, 2017; Wakusawa & Kawashima, 2016; Wang, 2013; Wang & Yu, 2017; Wang & Cai, 2016; Wang & Fan, 2007; Wang & Lv, 2019; Wei & Yang, 2023; Williams et al., 2006; Wu et al., 2019; Wu et al., 2023; Wu et al., 2017; Xiao et al., 2015; Xu & Cao, 2021; Ye & Gao, 2008; Ye & Zhang, 2007; Yu et al., 2022; Zeng, 2017; Zhang et al., 2017; Zhang & Huang, 2019; Zhang & Zhang, 2007; Zhang et al., 2015; Zhang & Ye, 2007; Zhao et al., 2021; Zhao & Jin, 2014; Zhong et al., 2023; Zhu & Xu, 2015; Zhu et al., 2023; Zhu, 2019; Zhuo & Fan, 2014) |
| **Not using fMRI technique** |
| (Cheng & Liu, 2017; Chouinard & Cummine, 2016; Dai et al., 2015; Du et al., 2022; Gold et al., 2010; Gu et al., 2006; Jiang et al., 2000; Jin & Liang, 2022; Li, Hu, Liu, Li, et al., 2017; C. Li et al., 2016; Li, Hu, Liu, Wang, et al., 2017; Li et al., 2013; Li, 2014a, 2014b; Y. Li et al., 2016; Lu et al., 2023; Lv et al., 2015; Shang et al., 2020; Shu et al., 2001; Song et al., 2021; Tang et al., 2021; Wang et al., 2019; Yankovitz et al., 2023; Zhang et al., 2020; Zhang, 2019; Zhang et al., 2011; Zhang et al., 2019; Zhou et al., 2021) |
| **Studies not related to rhetorical speech comprehension** |
| (author, 2017; Bai & Kong, 2019; F. Chen et al., 2017; Chen & Yang, 2014; L. Chen et al., 2017; Chen et al., 2023; Chen et al., 2019; Cheng et al., 2022; Cui, 2023; Guo et al., 2021; Guo et al., 2015; He, 2016; Huang & Wang, 2023; Huang et al., 2015; Huo et al., 2013; Ibrahim et al., 2021; Ji et al., 2006; Jia, 2007; Jia et al., 2018; Jing, 2021; Ke et al., 2009; Li et al., 2021; Li et al., 2010; J. Li et al., 2023; J. Li et al., 2014; X. Li et al., 2015; Li et al., 2013; X. Li et al., 2023; Li, Ao, et al., 2022; Y. Li et al., 2014; Li et al., 2019; Li, Zhang, et al., 2022; Y. Li et al., 2015; Lin et al., 2020; Liu et al., 2020; Liu et al., 2018; Liu et al., 2019; Liu & Miao, 2023; T. Liu et al., 2023; Liu et al., 2014; Z. Liu et al., 2023; Lu et al., 2018; Lu et al., 2023; Niu et al., 2016; Peng et al., 2017; Qiu et al., 2022; Su, 2010; Su & Lu, 2020; Su et al., 2020; Sun et al., 2020; Sun et al., 2014; C. Wang et al., 2021; Wang et al., 2016; Wang et al., 2017; Wang et al., 2009; Wang et al., 2022; Wang et al., 2020; X. Wang et al., 2021; Wang & Zou, 2018; Wei & Quan, 2008; Xing & Fang, 2013; Yang & Du, 2012; Yao et al., 2020; Yin et al., 2021; Yu et al., 2021, 2023; Yu et al., 2020; Zhang & Liu, 2018; Zhang et al., 2015; Zhang et al., 2020; Zhang & Wang, 2021; Zhao & Li, 2023; Zhao et al., 2021; Zheng et al., 2023; Zhou & Li, 2007; Zhou et al., 2022; Zhou et al., 2020; Zhou et al., 2021; Zou et al., 2010) |
| **Not ASD participants** |
| (Champagne-Lavau & Joanette, 2009; Chen et al., 2019; Chen et al., 2022; Cheng et al., 2018; Dan et al., 2020; Deschrijver & Palmer, 2020; Du et al., 2020; He et al., 2019; Hu & Shi, 2012; Hu et al., 2011; Huang et al., 2017; Ji et al., 2017; Lai et al., 2023; C. Liu et al., 2023; F. Liu et al., 2023; Liu et al., 2011; Liu et al., 2022; Z. Liu et al., 2023; Nakamura et al., 2022; Ning et al., 2023; Pallanti & Hollander, 2008; Ren, 2019; Shamay-Tsoory et al., 2006; Shibata et al., 2010; Simon et al., 2014; Thoma & Daum, 2006; Valles-Capetillo, Ibarra, Martinez, et al., 2022; Valles-Capetillo, Ibarra, & Giordano, 2022; Wakusawa et al., 2009; Wang et al., 2006; Wang et al., 2017; Wang et al., 2022; Wang et al., 2013; Wang et al., 2018; Xue et al., 2016; Yang et al., 2020; Zhou & Cheng, 2017; Zhou et al., 2017) |
| **Not including both ASD and TD groups** |
| (Mazza et al., 2022) |
| **Study using ROI analysis** |
| (Williams et al., 2013) |
| **No significant difference** |
| (Graves et al., 2022) |

**The assessment of included studies in this meta-analysis**

**Study Quality**

The modified version of the Newcastle-Ottawa scale (NOS) (mNOS) was used in previous studies (Costa et al., 2021; Gentili et al., 2019). There are 4 categories with 11 domains in the scale. The first category is the assessment of the study population selection, which was based on (1) the definition of the case; (2) the representativeness of the cases; (3) the selection of the controls; (4) the definition of the controls. The second category is the comparability between the experimental and control groups such as the age, gender and other variables like non-verbal intelligence quotient (IQ), full-scale IQ, native language and handedness. The third category assesses the quality of exposure which was based on (1) whether the two groups conduct the same experimental procedure, (2) whether dropouts was described and (3) whether it report the behavioural results. The fourth category is the assessment of the quality of statistical analysis: (1) whether a sufficient cluster-forming threshold was used the study; (2) whether an effective correction method was used to reduce the false positive results.

After the independent assessment of the included studies by the two authors, the evaluation agreement of the two authors was computed using kappa statistics in the online analysis software SPSSAU (<https://spssau.com/index.html>). Different opinions were resolved by discussion between the two authors. The results of the quality assessment were described in Table S2 and Table S3 (Supplementary materials).

**Table S5** Study-level risks of bias ratings after consensus and the kappa statistic agreement before consensus

| **Study** | **Selection** | | | |  | **Comparability** | |  | **Exposure** | | |  | **Statistical analysis** | |
| --- | --- | --- | --- | --- | --- | --- | --- | --- | --- | --- | --- | --- | --- | --- |
|  | **ACD** | **RoC** | **SoC** | **DoC** |  | **A & G** | **OV** |  | **SE** | **DR** | **BMC** |  | **p value < 0.001** | **FPC** |
| Chouinard et al. (2017) | – | ? | ? | + |  | + | + |  | – | ? | + |  | – | + |
| Colich et al. (2012) | + | ? | ? | + |  | + | + |  | + | ? | + |  | – | + |
| Kana et al. (2012) | + | + | + | ? |  | – | + |  | + | + | – |  | – | + |
| Kim et al. (2018) | + | + | + | + |  | + | + |  | + | + | + |  | – | + |
| Wang et al. (2006) | + | + | + | + |  | + | + |  | + | ? | + |  | – | + |
| Wang et al. (2007) | + | + | + | + |  | + | + |  | + | + | + |  | ? | + |
| Kappa agreement | 0.455 | 1 | 1 | 1 |  | 0.571 | 1 |  | 0.6 | 0.667 | 1 |  | 0.4 | 1 |
| Mean Kappa agreement | 0.81 |  |  |  |  |  |  |  |  |  |  |  |  |  |

Abbreviations: ACD, adequate case definition; RoC, representativeness of the cases; SoC, selection of controls; DoC, definition of controls; A & G, age & gender; OV, other variables; SE, same exposure; DR, dropout rate; BMC, behavioral manipulation check; FPC, false positive correction; +, low risk of bias; –, high risk of bias; ?, unclear risk of bias

| **Table S6** The results of (Mean) Kappa statistics | | | | | | |
| --- | --- | --- | --- | --- | --- | --- |
| **Analysis** | **Kappa** | **Standard Error**  **(Postulate null hypothesis)** | ***z*-value** | ***p*-value** | **Standard Error** | **95% CI** |
| author1 & author2 | 0.81 | 0.091 | 8.864 | 0.000** | 0.072 | 0.668 ~ 0.952 |
| ** p*<0.05 ** *p*<0.01 | | | | | | |
